# Nemonoxacin achieved a better symptomatic improvement and a prolonged interval to next exacerbation than moxifloxacin for outpatients with acute exacerbations of chronic obstructive pulmonary disease

**DOI:** 10.1038/s41598-023-44188-2

**Published:** 2023-10-07

**Authors:** Weiwei Meng, Huihui Zeng, Zhiqi Zhao, Ruoyan Xiong, Yan Chen, Zhuo Li

**Affiliations:** 1 Department of Pulmonary and Critical Care Medicine, The Second Xiangya Hospital, Central South University, Changsha, 410011 Hunan China; 2 Research Unit of Respiratory Disease, Central South University, Changsha, Hunan China; 3 Diagnosis and Treatment Center of Respiratory Disease, Changsha, Hunan China; 4 Center for Clinical Trails and Research, The Second Xiangya Hospital, Central South University, Changsha, 410011 Hunan China

**Keywords:** Diseases, Respiratory tract diseases, Chronic obstructive pulmonary disease

## Abstract

Nemonoxacin is a novel non-fluorinated quinolone with strong antibacterial efficacy, but data of its effect on acute exacerbations of chronic obstructive pulmonary disease (AECOPD) is rare. This study was conducted to compare the efficacy of oral nemonoxacin with moxifloxacin in AECOPD outpatients. In this retrospective observational study, a total of 101 AECOPD outpatients initially treated with nemonoxacin or moxifloxacin from July 2021 to March 2022 were enrolled. We collected COPD assessment test (CAT), Transition Dyspnea Indices (TDI) scores, and exacerbations information during 24 weeks follow-up from the electronic medical records. Kaplan–Meier curve was used to analyze the time to the next moderate/severe exacerbation. Compared to the moxifloxacin group, changes in CAT scores and TDI scores were significantly higher in the nemonoxacin group, and the nemonoxacin group also had a greater probability to reach the minimal clinically important difference of CAT (71.40% vs. 97.80%, *p* < 0.01) and TDI (40.50% vs. 60.00%, *p* < 0.05) at week 4. Despite no significant difference in the incidence of exacerbations between two groups, patients treated with nemonoxacin had a significantly prolonged time to next moderate/severe exacerbation than those with moxifloxacin (*p* < 0.05). Nemonoxacin achieved a better symptomatic improvement and a prolonged interval to next moderate/severe exacerbation for AECOPD outpatients.

## Introduction

Chronic obstructive pulmonary disease (COPD) places a significant health burden to China^1^, and acute exacerbations of COPD (AECOPD) cause substantial morbidity, mortality and marked reduction in lung function and quality of life^2^. Bacterial infection is a common reason for AECOPD, and possible causative pathogens varied from *Hemophilus influenza* to *Pseudomonas species*^2–5^. Thus, antibiotics are recommended in AECOPD patients with at least two cardinal symptoms, including increased purulence of sputum^2,3^. There was an abundance of evidence that antibiotic treatments could improve symptoms, reduce short-term mortality and the recurrence of exacerbations in AECOPD patients^6–8^. In addition, antibiotics could decrease the utilization of in-hospital health care for AECOPD outpatients^2^.

It is widely known that moxifloxacin and levofloxacin have been recommended to be used in the treatment of AECOPD^3,9,10^. Nemonoxacin is another innovative C8-methoxy non-fluorinated quinolone, which inhibits DNA synthesis through bacterial DNA gyrase, and is currently being used in clinical practice for treatments of common infectious diseases including community-acquired pneumonia (CAP), Clostridium difficile infections (CDIs) and so on^11,12^. In vitro activity studies demonstrated that nemonoxacin had a higher antibacterial activity against Gram-positive cocci and atypical pathogens than other quinolones (such as moxifloxacin and levofloxacin), while its activity against Gram-negative bacilli was similar to that of moxifloxacin and levofloxacin^13,14^. In addition, nemonoxacin also has a potent antimicrobial efficacy against antibiotic-resistant organisms like methicillin-resistant *Staphylococcus aureus* (MRSA) and penicillin-resistant *Streptococcus pneumoniae*^13–15^. Based on its non-inferiority antibacterial activity compared to other quinolones, nemonoxacin might be a preferable option for pulmonary infections.

Although lack of reliable evidence in randomized controlled trials (RCTs), Chinese consensus on AECOPD has recommended that nemonoxacin could be empirically used in the antimicrobial treatment of AECOPD^4^, owing to its broad antibacterial spectrum and potent antibacterial efficacy^15^. Moxifloxacin has been demonstrated in RCTs with good efficacy in AECOPD patients^10,16^. However, little is known about whether nemonoxacin could achieve the same clinical improvement in AECOPD patients as moxifloxacin. Therefore, this study was conducted to compare the efficacy of oral nemonoxacin with that of moxifloxacin for the treatment of outpatients with AECOPD.

## Methods

### Study design and population

We performed a retrospective observational study using the database of outpatients with COPD in the Second Xiangya Hospital of Central South University. Details of these patients’ populations have been presented elsewhere^17^. To compare the efficacy of oral nemonoxacin with moxifloxacin in outpatients with AECOPD, we enrolled patients from the database of outpatients with COPD in the Second Xiangya Hospital of Central South University between July 1, 2021 and March 30, 2022. The inclusion criteria were as follows: (1) AECOPD with more symptoms including increased in sputum purulence; (2) age ≥ 40 years; (3) patients received nemonoxacin (500 mg once daily for 6–9 days) or moxifloxacin (400 mg once daily for 6–9 days) as initial antimicrobial treatment regimens. Diagnosis and severity of COPD were confirmed by spirometry according to the Global Initiative for Chronic Obstructive Lung Disease (GOLD)^2^. Patients with other chronic respiratory diseases such as asthma, pulmonary fibrosis, or lung cancer were excluded; and patients with pneumonia, other infection diseases, or who had been treated with antibiotics within 4 weeks before enrollment were also excluded. All procedures performed in studies involving human participants were in accordance with the Declaration of Helsinki.

Extracted information included: age; sex; body mass index (BMI); educational level; residence; the smoking history; acute exacerbations (AEs) in the previous year; inhalers; COPD Assessment Test (CAT) scores at baseline and follow-up; Baseline Dyspnea Index (BDI) at baseline; Transition dyspnea index (TDI) at follow-up; spirometry obtained in the year prior to exacerbation ascertainment; laboratory results; incidence of exacerbations during 24 weeks follow-up and underlying comorbidities.

### Measurements

The CAT has been developed to provide a simple and reliable measure of disease-specific health status. The CAT consists of eight items (cough, phlegm, chest tightness, breathlessness, limited activities, confidence in leaving home, sleeplessness and energy) defined with contrasting adjectives. Item scores range from 0 to 5 points resulting in a CAT total score ranging from 0 to 40 points. The minimum clinically important difference (MCID) for CAT has been reported as a 2-point reduction^18,19^.

The Baseline and Transition Dyspnea Indices (BDI/TDI) provide measurements of breathlessness and of its impact on activities of daily living and 1-point increase is considered as the MCID for TDI^20,21^.

### Outcomes

Patients’ clinical notes were reviewed three times: at baseline, at Week 4 post-therapy (follow-up visit 1), and at Week 24 post-therapy (follow-up visit 2) to require details about: (1) changes from baseline in CAT scores and changes in TDI scores at Week 4 and Week 24; (2) the effectiveness of antibiotics based on the response rate of the MCID of CAT and TDI during 4 weeks and 24 weeks follow-up; (3) the incidence of exacerbations during 4 weeks and 24 weeks follow-up; (4) the time to first moderate/severe exacerbation during 24 weeks follow-up.

### Statistical analysis

Data are expressed as median values with interquartile ranges or means ± standard deviations. Statistical significance was reported using the Pearson’s χ^2^ test or Fisher’s exact test for categorical variables, and the Student’s t test, ANOVA or the Mann–Whitney U test for continuous variables. Kaplan–Meier curve with log-rank test was used to analyze the time to the first moderate/severe exacerbation during 24 weeks follow-up. A *p* value of < 0.05 was considered significant. All statistical analyses were performed with SPSS version 25.0 (SPSS Inc., Chicago, IL, USA).

## Ethics statement

The study was approved by the Ethics Committee of the Second Xiangya Hospital of Central South University with reference number 2018-040. All participants provided written informed consent.

## Results

### Demographics and clinical characteristics

This study included 101 AECOPD patients with mean age of 64.12 ± 7.51 years (range 52–86 years). The total number of male patients was 97 (96.0%) (Table 1). Of the 101 patients, 50 (49.5%) were treated with nemonoxacin and 51 (50.5%) were treated with moxifloxacin. The median number of exacerbations in the previous year was 1.00 (IQR: 0.00–2.00) for all patients. The median duration of initial antibiotic therapy was 6.00 (IQR: 6.00–9.00) days for all patients and the median FEV_1_% predicted was 47.60 (IQR: 37.17–58.40) (Table 1). No significant difference was found in the proportion of GOLD stages 3 and 4 between the nemonoxacin group and the moxifloxacin group (66.0% vs. 45.1%, *p* = 0.083). There was no significant difference in inhalers and other medicines use. No differences were found at baseline characteristics and laboratory results [blood routine, serum C-reactive protein (CRP) and erythrocyte sedimentation rate (ESR)] between the two groups (Tables 1, 2).

**Table 1:** Demographic and clinical characteristics of all patients.

Characteristics	Total (*n* = 101)	Nemonoxacin group (*n* = 50)	Moxifloxacin group (*n* = 51)	*p *value
Age, years*	64.12 ± 7.51	65.08 ± 8.19	63.18 ± 6.73	0.385
Male	97 (96.0)	49 (98.0)	48 (94.1)	0.317
BMI, kg/m^2^ *	21.68 ± 3.47	21.40 ± 3.60	21.95 ± 3.35	0.408
Educational level				0.864
Primary and below	33 (32.7)	16 (32.0)	17 (33.3)	
Secondary	40 (39.6)	21 (42.0)	19 (37.3)	
High school	17 (16.8)	7 (14.0)	10 (19.6)	
University and above	11 (10.9)	6 (12.0)	5 (9.8)	
History of smoking				0.722
Current smoker	40 (39.6)	18 (36.0)	22 (43.1)	
Ex-smoker	51 (50.5)	28 (56.0)	23 (45.1)	
Never smoker	10 (9.9)	4 (8.0)	6 (11.8)	
Duration of antibiotic therapy, days	6.00 (6.00–9.00)	6.00 (6.00–9.00)	6.00 (6.00–6.00)	0.142
AEs in the previous year	1.00 (0.00–2.00)	1.00 (0.00–2.00)	1.00 (0.00–2.00)	0.344
CAT score*	20.16 ± 5.02	20.71 ± 5.81	19.60 ± 4.07	0.297
BDI total score	7.00 (5.00–8.00)	6.00 (5.00–8.00)	7.00 (4.75–9.00)	0.189
Lung function				
Post-BD FEV_1_ (% pred)	47.60 (37.17–58.40)	43.70 (34.27–55.80)	48.55 (37.85–59.50)	0.307
Post-BD FEV_1_/FVC (%)	43.23 (34.00–49.07)	42.73 (34.76–47.84)	43.61 (34.00–51.32)	0.643
Inhalers				0.467
LAMA	6 (5.9)	2 (4.0)	4 (7.8)	
ICS + LABA	6 (5.9)	2 (4.0)	4 (7.8)	
LABA + LAMA	34 (33.7)	20 (40.0)	14 (27.5)	
ICS + LABA + LAMA	55 (54.5)	26 (52.0)	29 (56.9)	
Comorbidities				
Hypertension	34 (33.7)	20 (40.0)	14 (27.5)	0.216
Diabetes mellitus	6 (5.9)	3 (6.0)	3 (5.9)	1.000
Cardiovascular disease	16 (15.8)	9 (18.0)	7 (13.7)	0.589
Chronic heart failure	3 (3.0)	1 (2.0)	2 (3.9)	1.000
Myocardial infarction	1 (1.0)	1 (2.0)	0 (0.0)	1.000
Peripheral arterial disease	2 (2.0)	1 (2.0)	1 (2.0)	1.000
Chronic kidney disease	6 (5.9)	3 (6.0)	3 (5.9)	1.000

Data are presented as n (%) or median (IQR) unless otherwise stated. *Data are presented as the mean (SD).

*AECOPD* acute exacerbations of chronic obstructive pulmonary disease, *BMI* body mass index, *AEs* acute exacerbations, *LAMA* long-acting muscarinic receptor antagonist, *ICS* inhaled corticosteroids, *LABA* long-acting beta-adrenoceptor agonist, *CAT* COPD Assessment Test, *mMRC* modified Medical Research Council, *BDI* baseline dyspnea index, *GOLD* global initiative for chronic obstructive lung disease, *BD* bronchodilator, *FEV*_*1*_ forced expiratory volume in 1 s, *FVC* forced vital capacity.

**Table 2:** Results of clinical blood routine and serological examinations in all patients.

Characteristics	Nemonoxacin group (*n* = 41)	Moxifloxacin group (*n* = 27)	*p* value
Blood routine			
WBC (^^^10^9^/L)	7.03 (5.04–8.64)	7.80 (6.38–8.96)	0.080
RBC (^^^10^12^/L)	4.66 (4.31–5.01)	4.79 (4.57–5.18)	0.116
HGB (g/L)	141.00 (122.50–151.00)	144.00 (139.00–152.00)	0.111
PLT (^^^10^9^/L)	209.00 (176.50–271.00)	247.00 (192.00–290.00)	0.226
NEUT# (^^^10^n^/L)	4.72 (3.01–6.44)	5.16 (4.26–5.98)	0.361
NEUT%	66.80 (60.70–74.50)	66.80 (58.80–72.30)	0.726
CRP (mg/L)	3.55 (2.15–10.80)	4.50 (2.33–8.98)	0.758
ESR (mm/h)	11.50 (7.25–40.25)	10.00 (5.00–16.00)	0.245

Data are presented as median (IQR).

*IQR* interquartile range, *WBC* white blood cell, *RBC* red blood cell, *HGB* hemoglobin, *PLT* platelet count, *NEUT* neutrocyte, *CRP* C-reactive protein, *ESR* erythrocyte sedimentation rate.

### Improvement of CAT

Overall, CAT total scores at week 4 and week 24 decreased significantly when compared with the scores at baseline in both the nemonoxacin and moxifloxacin groups (Table S1). However, the decline of CAT total scores in the nemonoxacin group was significantly greater than that in the moxifloxacin group either at week 4 or week 24 [− 12.00 (IQR: − 16.00 to − 6.00) vs. − 9.00 (IQR: − 16.00 to 0.00), *p* < 0.05; − 13.50 (IQR: − 20.75 to − 8.75) vs. − 10.50 (IQR: − 15.25 to − 6.50), *p* < 0.05, respectively, Table 3].

**Table 3:** Changes of CAT scores during 4 weeks and 24 weeks follow-up.

Characteristics	Up to 4 weeks			Up to 24 weeks		
	Moxifloxacin group (*n* = 42)	Nemonoxacin group (*n* = 45)	*p* value	Moxifloxacin group (*n* = 38)	Nemonoxacin group (*n* = 41)	*p* value
CAT score	9.00 (6.50–14.00)	9.50 (6.00–13.00)	0.698	6.50 (5.00–9.00)	7.00 (5.00–10.25)	0.414
∆CAT score	− 9.00 (− 16.00 to 0.00)	− 12.00 (− 16.00 to − 6.00)	**< **0.05	− 10.50 (− 15.25 to − 6.50)	− 13.50 (− 20.75 to − 8.75)	**< **0.05
ΔCAT individual item score						
Cough	− 1.00 (− 2.00 to 0.00)	− 2.00 (− 2.00 to − 1.00)	0.062	− 1.00 (− 2.00 to 0.00)	− 2.00 (− 3.00 to − 1.00)	**< **0.05
Phlegm	− 1.00 (− 2.00 to 0.00)	− 2.00 (− 2.00 to − 1.00)	**< **0.05	− 1.00 (− 2.00 to 0.00)	− 2.00 (− 3.00 to − 1.00)	**< **0.05
Chest tightness	− 1.00 (− 2.00 to 0.00)	− 2.00 (− 3.00 to − 1.00)	**< **0.01	− 2.00 (− 3.00 to 0.00)	− 2.00 (− 3.00 to − 1.00)	0.241
Breathlessness	− 1.00 (− 3.00 to 0.00)	− 2.00 (− 3.00 to − 1.00)	0.101	− 1.00 (− 2.00 to 1.00)	− 1.00 (− 2.00 to − 1.00)	0.122
Limited activities	− 2.00 (− 2.00 to − 0.75)	− 2.00 (− 3.00 to − 1.00)	0.396	− 2.00 (− 3.00 to 0.00)	− 2.00 (− 3.00 to − 1.00)	0.617
Confidence in leaving home	− 1.00 (− 2.00 to 1.00)	− 2.00 (− 3.00 to − 0.50)	**< **0.05	− 1.00 (− 2.00 to 0.00)	− 2.00 (− 3.00 to − 1.00)	**< **0.01
Sleeplessness	− 0.50 (− 2.00 to 0.00)	− 1.00 (− 2.00 to 0.00)	0.123	− 2.00 (− 2.00 to 0.00)	− 2.00 (− 3.00 to − 1.00)	0.181
Energy	− 1.00 (− 2.00 to 0.00)	− 1.00 (− 1.00 to 1.00)	0.169	− 2.00 (− 2.00 to − 1.00)	− 2.00 (− 3.00 to − 1.00)	0.660
ΔCAT pulmonary item score	− 5.50 (− 8.25 to − 1.00)	− 8.00 (− 10.00 to − 4.50)	**< **0.01	− 4.00 (− 7.00 to − 3.00)	− 7.00 (− 10.00 to − 5.00)	**< **0.01
ΔCAT extra-pulmonary item score	− 4.50 (− 7.00 to 0.00)	− 5.00 (− 7.50 to − 2.00)	0.236	− 6.50 (− 8.00 to − 4.50)	− 7.00 (− 10.50 to − 5.00)	0.095

Data are presented as median (IQR).

*IQR* interquartile range, *AECOPD* acute exacerbations of chronic obstructive pulmonary disease, *CAT* COPD assessment test.

**Figure 1:**
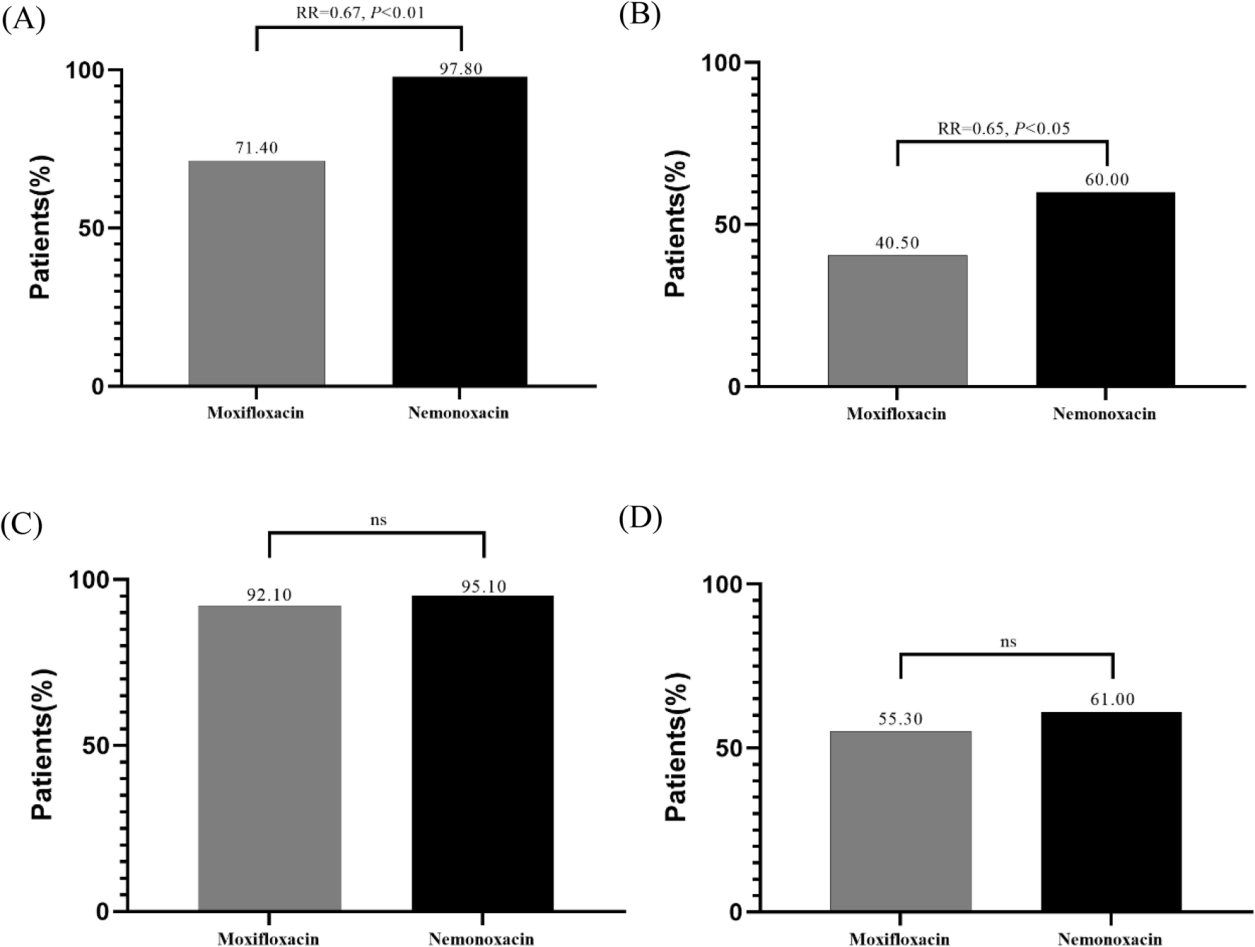
Comparison of the MCID response rate in CAT scores and TDI scores between the moxifloxacin group and the nemonoxacin group. (**A**) MCID response rate in CAT scores after 4 weeks; (**B**) MCID response rate in TDI scores after 4 weeks; (**C**) MCID response rate in CAT scores after 24 weeks; (**D**) MCID response rate in TDI scores after 24 weeks. *CAT* COPD assessment test, *TDI* transition dyspnea indices, *MCID* minimal clinically important differences, *RR* risk ratio.

The nemonoxacin group had a higher proportion of patients reaching the MCID of CAT than moxifloxacin at Week 4 (97.80% vs. 71.40%, *p* < 0.01, Fig. 1A). However, there was no significant difference in the proportion of patients reaching the MCID of CAT between groups at Week 24 (95.10% vs. 92.10%, Fig. 1C).

Individual item scores for respiratory items were significantly improved in the nemonoxacin group than that in the moxifloxacin group at week 4, such as ‘phlegm’ [− 2.00 (IQR: − 2.00 to − 1.00) vs. − 1.00 (IQR: − 2.00 to 0.00), *p* < 0.05, Table 3] and ‘chest tightness’ [− 2.00 (IQR: − 3.00 to − 1.00) vs. − 1.00 (IQR: − 2.00 to 0.00), *p* < 0.01, Table 3]. Moreover, individual item scores for items ‘cough’ [− 2.00 (IQR: − 3.00 to − 1.00) vs. − 1.00 (IQR: − 2.00 to 0.00), *p* < 0.05, Table 3] and ‘phlegm’ [− 2.00 (IQR: − 3.00 to − 1.00) vs. − 1.00 (IQR: − 2.00 to 0.00), *p* < 0.05, Table 3] were also significantly improved in the nemonoxacin than moxifloxacin groups at week 24.

**Figure 2:**
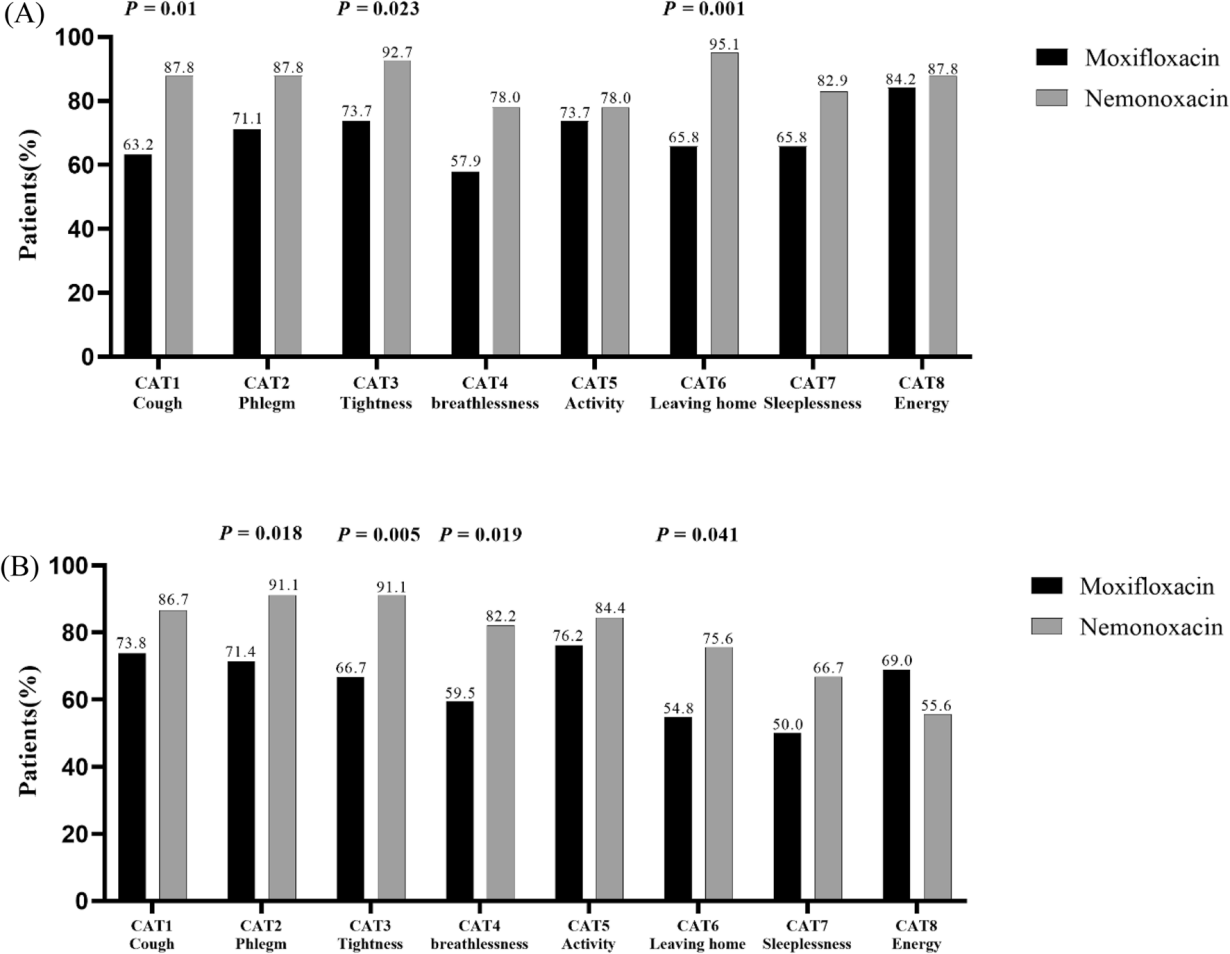
Percentage of patients reporting an improvement in CAT items by patients reporting a decline (≥ 1 point) or an improvement (≤ − 1 point) in CAT items following moxifloxacin or nemonoxacin therapy (**A**) during 4 weeks and (**B**) 24 weeks follow-up. *CAT* COPD assessment test.

Accordingly, the percentages of patients reporting an improvement (≤ − 1 point) in eight CAT items also showed significant differences after moxifloxacin and nemonoxacin therapy. Compared with moxifloxacin, the percentages of an improvement in CAT items ‘phlegm’, ‘chest tightness’, ‘breathlessness’ and ‘confidence in leaving home’ were higher in the nemonoxacin group at week 4 (Table S2, Fig. 2A). At week 24, the percentages of reporting an improvement in CAT items ‘cough’, ‘chest tightness’ and ‘confidence in leaving home’ were higher after nemonoxacin treatment (Table S2, Fig. 2B).

### Improvement of TDI

Dyspnea, evaluated using TDI total scores, improved significantly in the nemonoxacin group than that in the moxifloxacin group at week 4 [3.00 (IQR: 0.00–5.25) vs. 1.00 (IQR: − 5.00 to 6.00), *p* < 0.05, Table 4]. And the TDI effort scores were also significantly higher in the nemonoxacin group [0.00 (IQR: 0.00–2.00) vs. 0.00 (IQR: − 3.00 to 2.00), *p* < 0.05, Table 4] at week 4. However, no significant between-group differences in TDI scores were observed at week 24.

**Table 4:** Changes of TDI scores during 4 weeks and 24 weeks follow-up.

Characteristics	Up to 4 weeks			Up to 24 weeks		
	Moxifloxacin group (*n* = 42)	Nemonoxacin group (*n* = 45)	*p* value	Moxifloxacin group (*n* = 38)	Nemonoxacin group (*n* = 41)	*p* value
TDI						
Total score	1.00 (− 5.00 to 6.00)	3.00 (0.00–5.25)	**< **0.05	1.00 (− 2.00 to 2.25)	1.00 (− 1.00 to 3.25)	0.291
Functional impairment	0.00 (− 2.00 to 3.00)	2.00 (0.00–3.00)	0.197	0.00 (− 1.00 to 1.00)	0.00 (− 1.00 to 1.00)	0.822
Magnitude of task	0.00 (− 2.00 to 2.00)	0.00 (0.00–2.00)	0.186	0.00 (0.00–1.00)	1.00 (0.00–1.00)	0.129
Magnitude of effort	0.00 (− 3.00 to 2.00)	0.00 (0.00–2.00)	**< **0.05	1.00 (− 1.00 to 1.00)	1.00 (− 1.00 to 1.00)	0.408

Data are presented as medians (IQR).

*IQR* interquartile range, *AECOPD* acute exacerbations of chronic obstructive pulmonary disease, *TDI* transition dyspnea indices.

Compared to the moxifloxacin group, the nemonoxacin group was more likely to reach the MCID of TDI (40.50% vs. 60.00%, *p* < 0.05, Fig. 1B) at Week 4. While the proportion of patients reaching the MCID of TDI was similar between groups at Week 24 (55.30% vs. 61.00%, Fig. 1D).

### Acute exacerbations of COPD

There were no deaths during 24 weeks follow-up. The median number of exacerbations during 24 weeks follow-up was 0.00 (IQR: 0.00–1.00). The incidence rate of exacerbations was 5.7% during 4 weeks follow-up, and was 36.7% during 24 weeks follow-up, indicating that the study population had a high probability to experience next exacerbation. Between the nemonoxacin and the moxifloxacin groups, the incidence rates of total exacerbations (2.2% vs. 9.5% at week 4, 31.7% vs. 42.1% at week 24, Table 5) and moderate/severe exacerbations (2.2% vs. 9.5% at week 4, 24.4% vs. 42.1% at week 24, Table 5) were similar.

**Table 5:** Exacerbations of patients during 4 weeks and 24 weeks follow-up.

Characteristics	Up to 4 weeks		Up to 24 weeks	
	Moxifloxacin group (*n* = 42)	Nemonoxacin group (*n* = 45)	Moxifloxacin group (*n* = 38)	Nemonoxacin group (*n* = 41)
AEs				
No	38 (90.5)	44 (97.8)	22 (57.9)	28 (68.3)
Yes	4 (9.5)	1 (2.2)	16 (42.1)	13 (31.7)
≥ 2	–	–	4 (10.5)	5 (12.2)
Moderate/severe AEs				
No	38 (90.5)	44 (97.8)	22 (57.9)	31 (75.6)
Yes	4 (9.5)	1 (2.2)	16 (42.1)	10 (24.4)

Data are presented as numbers (%).

*AEs* acute exacerbations.

**Figure 3:**
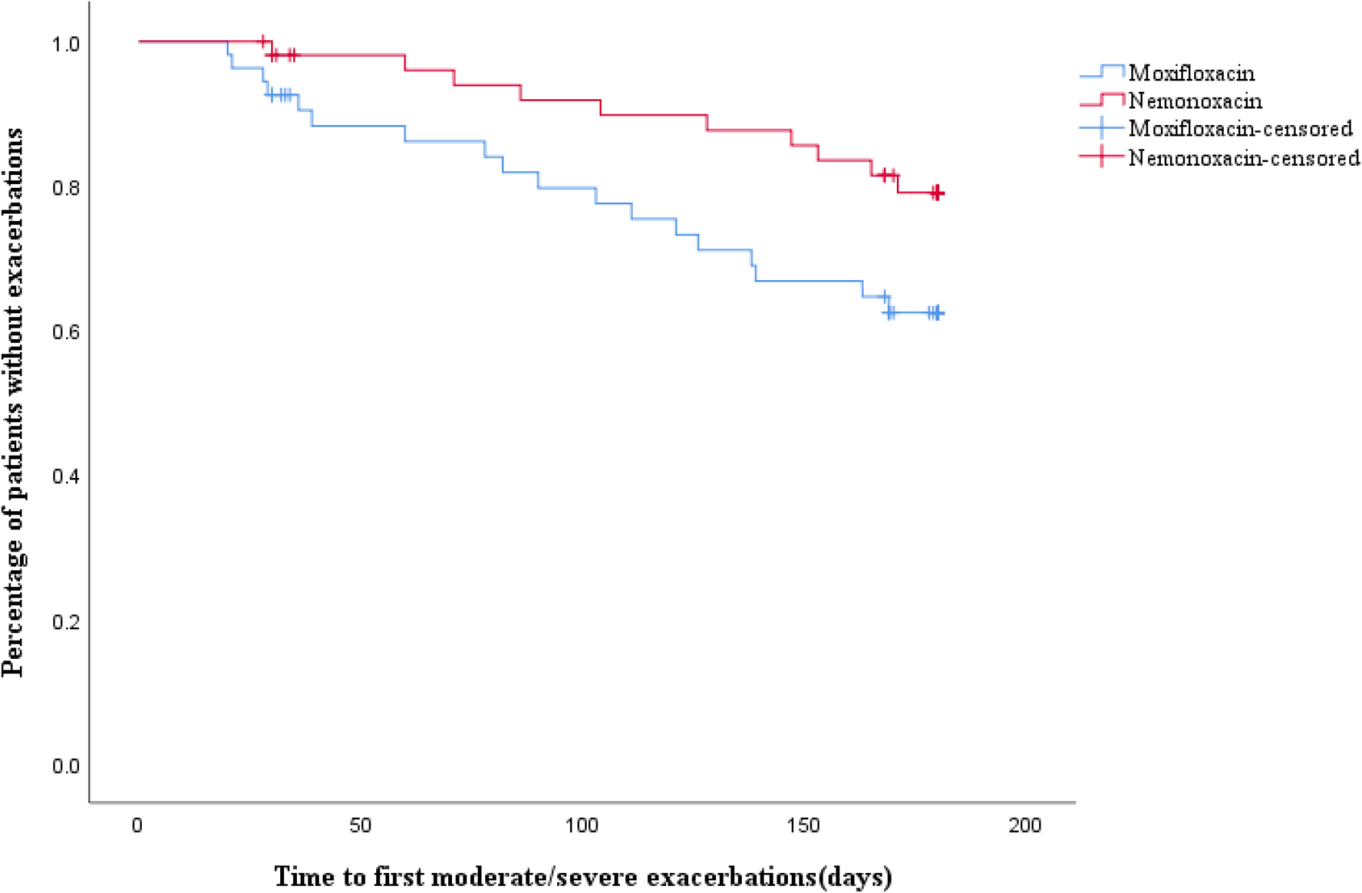
Kaplan–Meier time-to-event plot and log-rank test for the time to first moderate/severe exacerbation during 24 weeks follow-up among the moxifloxacin group and nemonoxacin groups.

Besides that, the median time to the next exacerbation of the whole population was 96.50 days and there was no significant difference in the median time to next exacerbation between the nemonoxacin and moxifloxacin groups (116.00 days vs. 86.00 days). Interestingly, the Kaplan–Meier curve found that patients treated with nemonoxacin had a significantly prolonged time to next moderate/severe exacerbation (*p* < 0.05, Fig. 3), compared to moxifloxacin.

## Discussion

This retrospective observational study compared the efficacy of oral nemonoxacin with that of moxifloxacin for the treatment of AECOPD outpatients. Moxifloxacin is a well-accepted antibiotic in the treatment of AECOPD^2,10^, and nemonoxacin is also slightly recommended in Chinese consensus on AECOPD without abundance data^4^. However, litter is known about whether there are differences between the two quinolones in the treatment of AECOPD in real world. This study found that initial treatment with nemonoxacin in AECOPD outpatients could achieve a better symptomatic improvement assessed by CAT and TDI scales, and a longer interval to next moderate/severe exacerbation in comparison with moxifloxacin.

It is widely known that most incidences of exacerbations in COPD are associated with frequent bacterial infections^5,22^. Thus, antibiotics play a pivotal role in the treatment regimens. The MAESTRAL study found that moxifloxacin resulted in significantly high clinical success rates and high bacterial eradication for outpatients with moderate-to-severe AECOPD with a suspected bacterial etiology^10^. Llor et al.^23^ reported that amoxicillin/clavulanate is more effective and significantly prolongs the time to the next exacerbation in moderate exacerbations of mild-to-moderate COPD compared with placebo. These clinical trials confirmed clinical (such as symptomatic improvements, fewer exacerbations, etc.) or bacteriological (bacterial eradication) superiority of antibiotics in the treatment of AECOPD. The CAT and BDI/TDI scales are frequently used measurement tools to evaluate patients’ symptoms in clinical practice^24^. Several studies recently demonstrated that monitoring the changes of the CAT scores during an AECOPD could assess the treatment response^25,26^. This study evaluated the response to antibiotics in AECOPD outpatients by tracking CAT and TDI score changes. In line with previous studies, our results showed significantly improved symptom scores after nemonoxacin and moxifloxacin treatment. It indicated the good efficacy of both nemonoxacin and moxifloxacin in AECOPD outpatients, which may be explained by their effective antibacterial activity against common pathogens causing exacerbations^13^.

Moreover, this study observed more obvious improvements in total CAT and TDI scores of AECOPD outpatients treated with nemonoxacin rather than moxifloxacin during follow-up, indicating that nemonoxacin may be better than moxifloxacin in terms of symptom reduction during the treatment of exacerbations. Chinese consensus on AECOPD demonstrated that the three most common pathogens are *H. influenzae*, *Moraxella catarrhalis*, and *S. pneumoniae* respectively in AECOPD^4^. One study in Canada reported that nemonoxacin was at least fourfold more active than moxifloxacin against most Gram-positive cocci^13^. Liu et al.^27^ found that nemonoxacin had stronger antibacterial activity against *S. pneumoniae*, *S. aureus* (including MRSA), *H. influenzae* and *Klebsiella pneumoniae* strains than levofloxacin in the treatment of CAP. What’s more, in vivo and in vitro studies demonstrated that nemonoxacin had less minimum inhibitory concentrations (MIC) than moxifloxacin and levofloxacin, against some Gram-positive or negative bacteria^11,28^. These studies suggest that nemonoxacin might be a promising choice for bacterial infections with better antibacterial activity than other quinolones, and could explain a better clinical outcome from nemonoxacin than moxifloxacin in our results.

Although microbiological eradication rates are generally considered to be an important indicator for effective antibacterial therapy^29^, the effectiveness of antibiotics for outpatients cannot be determined only by this factor because sputum culture is sometimes limited in the outpatient setting due to time-consuming (at least 2 days) and technical reasons^8,30^. The evidence based on sputum culture results suggests that purulence of sputum is the most sensitive indicator to judge the elevated bacterial load of the lower respiratory tract^4,30^. Accordingly, the more significant improvement in individual CAT item ‘phlegm’ in nemonoxacin-treated patients could also confirm the better efficacy in symptomatic improvements and lower bacterial burden in the nemonoxacin group than the moxifloxacin group.

It should be pointed out that our study population was at high risk of experiencing next exacerbation, with 36.7% of the relapse rate and 96.50 days of the median time to next exacerbation during 24 weeks follow-up. Previous studies indicated that the recurrence is associated with persistent airway and systemic inflammation and thereby structural airway damage^31^. One possible explanation of this persistent inflammation is incomplete resolution of airway bacterial infections. Despite no significant differences in the relapse rates of exacerbations between two groups, nemonoxacin had a significantly longer time to the next moderate/severe exacerbation. Evidence from clinical trials indicated that COPD patients with improved health status or lower CAT scores had a lower likelihood of exacerbation^24^. Based on the potent strong efficacy against pathogens of nemonoxacin, this result may indicate more effective bacterial eradication and better health status in patients treated with nemonoxacin.

The study also has some limitations. First, this was a single-center, retrospective, observational study with a small sample in China, which limits its generalizability to other populations. Second, we lack bacteriological assessment, which precludes a correlation with the clinical outcomes. A further prospective clinical trial with larger sample is required.

## Conclusion

This retrospective observational study indicated that oral nemonoxacin was superior to oral moxifloxacin in rapid symptom improvement for initial antimicrobial treatment of outpatients with AECOPD. As an antibiotic with a broad antibacterial spectrum, strong antibacterial efficacy, and less tendency to being resistant, nemonoxacin may be a useful option for the treatment of AECOPD outpatients.

### Supplementary Information

Supplementary Information.

## Data Availability

The datasets presented in this article are not readily available because “The raw data supporting the conclusions of this article will be made available with reasonable request.” Requests to access the datasets should be directed to “chenyan99727@csu.edu.cn”.
