# Supplementary material for: Nemonoxacin achieved a better symptomatic improvement and a prolonged interval to next exacerbation than moxifloxacin for outpatients with acute exacerbations of chronic obstructive pulmonary disease
Source: Sci Rep. 2023 Oct 7;13:16954. doi: 10.1038/s41598-023-44188-2 (PMC10560244; doi:10.1038/s41598-023-44188-2)
Supplement: Supplementary file 1 — Supplementary Information. [file 41598_2023_44188_MOESM1_ESM.pdf]

Supplementary File

1) Table S1. Changes in total CAT scores before and after antibiotics therapy.

2) Table S2. Percentage of patients reporting an improvement (≤ -1 point) in CAT individual items and proportion of patients achieving MCID of CAT and TDI.

Table S1 Changes in total CAT scores before and after antibiotics therapy.

Characteristics	Subgroup	Before therapy	After therapy	*p* - value
**4 weeks**	**Moxifloxacin group (*n* = 42)**	18.50 (16.00 - 21.25)	9.00 (6.00 - 12.50)	< 0.001
	**Nemonoxacin group (*n* = 45)**	23.00 (16.50 - 27.50)	9.00 (6.00 - 12.50)	< 0.001
**24 weeks**	**Moxifloxacin group (*n*= 38)**	17.00 (14.75 - 21.00)	7.00 (5.00 - 9.00)	< 0.001
	**Nemonoxacin group (*n* = 41)**	22.00 (16.00 - 29.50)	7.00 (5.00 - 9.50)	< 0.001

Notes: Data are presented as median (IQR).

Legend: IQR = interquartile range; CAT = COPD Assessment Test.

Table S2 Percentage of patients reporting an improvement (≤ -1 point) in CAT individual items and proportion of patients achieving MCID of CAT and TDI.

Characteristics	Up to 4 weeks			Up to 24 weeks		
	Moxifloxacin group (*N* = 42)	Nemonoxacin group (*N* = 45)	*p* -value	Moxifloxacin group (*N* = 38)	Nemonoxacin group (*N* = 41)	*p* -value
**CAT individual item**						
**Cough**	31 (73.8)	39 (86.7)	0.131	24 (63.2)	36 (87.8)*	<0.05
**Phlegm**	30 (71.4)	41 (91.1)*	<0.05	27 (71.1)	36 (87.8)	0.064
**Chest Tightness**	28 (66.7)	41 (91.1)*	<0.01	28 (73.7)	38 (92.7)*	<0.05
**Breathlessness**	25 (59.5)	37 (82.2)*	<0.05	22 (57.9)	32 (78.0)	0.054
**Limited activities**	32 (76.2)	38 (84.4)	0.332	28 (73.7)	32 (78.0)	0.650
**Confidence in leaving home**	23 (54.8)	34 (75.6)*	<0.05	25 (65.8)	39 (95.1)**	<0.01
**Sleeplessness**	21 (50.0)	30 (66.7)	0.115	25 (65.8)	34 (82.9)	0.080
**Energy**	29 (69.0)	25 (55.6)	0.195	32 (84.2)	36 (87.8)	0.645
**MCID of CAT**			<0.01			0.667
**Yes**	30 (71.4)	44 (97.8)**		35 (92.1)	39 (95.1)	
**No**	12 (28.6)	1 (2.2)**		3 (7.9)	2 (4.9)	
**MCID of TDI**			<0.05			0.607
**Yes**	17 (40.5)	27 (60.0)*		21 (55.3)	25 (61.0)	
**No**	25 (59.5)	18 (40.0)*		17 (44.7)	16 (39.0)	

Notes: Data are presented as n (%).

Legend: CAT = COPD Assessment Test; TDI = Transition Dyspnea Indices; MCID = Minimal clinically important difference.

$^{*}P < 0.05$, $^{**}P < 0.01$.
